# Inside the November 2024 Issue 

**DOI:** 10.24908/pocus.v9i2.18344

**Published:** 2024-11-15

**Authors:** Benjamin T Galen

**Affiliations:** 1 Department of Medicine, Albert Einstein College of Medicine and Montefiore Medical Center Bronx, NY USA

**Keywords:** Commentary, POCUS Journal

Dear Readers,

We are thrilled to bring you the second issue of the ninth volume of POCUS Journal. Published since 2016, POCUS Journal is the only multi-disciplinary, peer-reviewed, POCUS-focused journal that is free for authors and readers alike. 

This issue features a variety of POCUS articles from around the world: from North America, South America, Europe, Asia, and even Australia. In addition to novel cases with striking POCUS images and video clips, this issue boasts a great deal of educational research at all levels: **Danila et al. (page 80)** report on their study of how POCUS can augment a medical student preclinical cardiovascular course while **Piro** **et al. (page 93)** studied the impact of just-in-time POCUS curriculum on internal medicine residents; **Janjigian** **et al. (page 109)** studied the longitudinal impact of a faculty POCUS training course. 

POCUS is a powerful tool with a wide range of applications: for example, it can be used both to diagnose musculoskeletal conditions (**Doblinger** **et al. page 15 **and **Mack et al. page 27**) and to treat musculoskeletal conditions (**Bowling et al page 12**, and **McCreary et al. page 30**). POCUS can also be used to predict heart failure readmission (**Malagón** **et al. page 125**). We are so excited to showcase pivotal POCUS scholarship like this in the November issue. 

I hope you enjoy this issue of POCUS Journal as much as I have. A huge thank you to our authors, editorial board, and publisher. 

Sincerely ,

Benjamin T. Galen, MD

Department of Medicine, Albert Einstein College of Medicine and Montefiore Medical Center, Bronx, NY, USA

Editor in– Chief, 

POCUS Journal 

**Figure 1:**
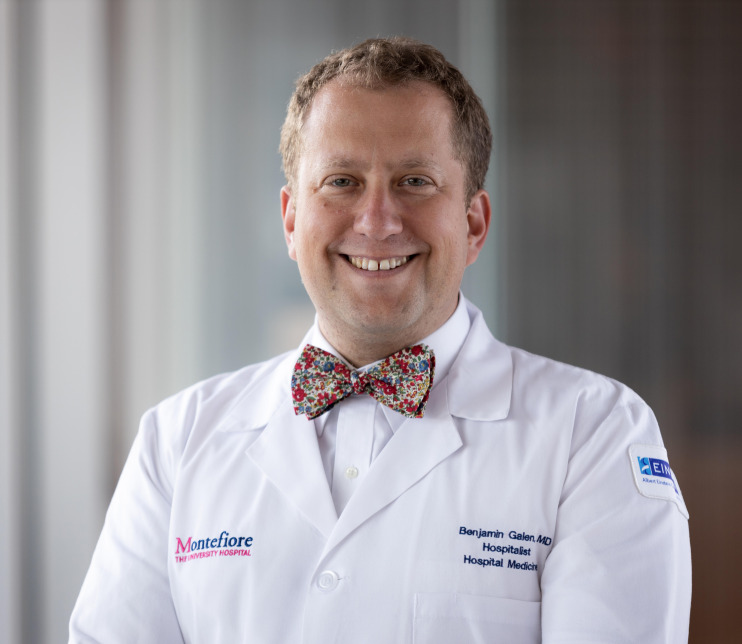
Benjamin T. Galen, Editor in Chief, POCUS Journal

